# Association of *CILP2* and *ACE* Gene Polymorphisms with Cardiovascular Risk Factors in Slovak Midlife Women

**DOI:** 10.1155/2013/634207

**Published:** 2013-11-20

**Authors:** Lenka Luptáková, Dominika Benčová, Daniela Siváková, Marta Cvíčelová

**Affiliations:** Department of Anthropology, Faculty of Natural Sciences, Comenius University, Mlynska Dolina, 842 15 Bratislava, Slovakia

## Abstract

The aim of this study is to assess the association of two polymorphisms, the cartilage intermediate layer protein 2 (*CILP2*) G/T and angiotensin converting enzyme (*ACE*) I/D, with blood pressure and anthropometrical and biochemical parameters related to the development of cardiovascular disease. The entire study sample comprised 341 women ranging in age from 39 to 65 years. The *CILP2* genotypes were determined by PCR-RFLP and the *ACE* genotypes by PCR. The Bonferroni pairwise comparisons showed the effect of the *CILP2* genotype on high density lipoprotein cholesterol (HDL-C), low density lipoprotein cholesterol (LDL-C), apolipoprotein B (apoB), apoB-to-apoA1 ratio, the total cholesterol (TC)-to-HDL-C ratio, non-HDL-C, and the LDL-C-to-HDL-C ratio (*P* < 0.05). Here, higher mean levels of HDL-C and lower mean levels of the remaining above mentioned lipid parameters were registered in the GT/TT genotype carriers than in GG carriers. Statistically significant association was identified between the *ACE* genotype and the following parameters: TC, LDL-C, and non-HDL-C (*P* < 0.05). The II genotype can lower serum level of TC (*B* = 0.40), LDL-C (*B* = 0.37), and non-HDL-C levels. The results of this study suggest that the minor T allele of *CILP2* gene and I allele of *ACE* gene have a protective effect against elevated serum lipid and lipoprotein levels.

## 1. Introduction

Increased blood lipid and lipoprotein levels, low HDL cholesterol concentration, glucose intolerance, hypertension, and obesity have emerged as some of the most serious public health concerns in recent decades. These variables are closely related to a number of pathological disorders including cardiovascular disease (CVD). Although recent increases in CVD risk factors often reflect lifestyle changes, genetic factors also play a substantial role. Genome-wide association studies have revealed the association of DNA polymorphisms in both the *CILP2* gene (cartilage intermediate layer protein) and the *ACE* gene (angiotensin converting enzyme) with CVD risk factors [1–3].

The *CILP2* gene codes for a noncollagenous protein recently isolated from human articular cartilage. Kathiresan et al. [1] reported that an intergenic region between *CILP2* and *PBX4* (pre-B-cell leukaemia homeobox 4) located in chromosome 19p13 is associated with concentrations of low-density lipoprotein cholesterol (LDL-C) and triglycerides (TG). The minor allele at SNP rs16996148 was associated with lower concentrations of both LDL-C and TG. In addition, Tai et al. [4] examined the association between this polymorphism and elevated high density lipoprotein cholesterol (HDL-C) levels in an Asian Malay population. In Slovakia, Rašlová et al. [5] identified an association between *CILP2* allele and atherogenic index log (TG-to-HDL-C ratio) in Slovak women and FER_HDL_ (cholesterol esterification rate in HDL plasma) in both genders. Genetic analysis has also highlighted a significant association between polymorphisms in the *CILP* gene and osteoarthritis progression [6].

Angiotensin converting enzyme (*ACE*) plays an important role in the pathophysiology of CVD. Although *ACE* is mainly localized in the endothelium of blood vessels, especially in the pulmonary circulation, it is also found in epithelial cells, in mononuclear blood vessels, and in macrophages [7]. *ACE* is a key enzyme in the body's renin-angiotensin system (RAS), modulating the synthesis of angiotensin II and inactivation of bradykinin. The *ACE* gene has an insertion/deletion (I/D) polymorphism, with the D allele associated with higher *ACE* levels [8, 9]. The deletion allele (D) has been implicated in the pathogenesis of a variety of CVD risk factors and disorders including hypertension and diabetes mellitus [2], metabolic syndrome [3], coronary heart disease [10], and myocardial infarction [11]. However, the associations between the *ACE* I/D polymorphism and most of these conditions were found to be inconsistent in different population investigations, with controversial results dependent on ethnic background, gender, or the individual's analyzed health status [12, 13]. Furthermore, meta-analysis indicated that *ACE* D allele is associated with population age distribution and longevity in the majority of European populations [14]. 

The aim of this study is to examine if genetic variants in both *CILP2* and *ACE* genes are associated with different anthropometrical, biochemical parameters and blood pressure in a nonclinical study sample of midlife women from Slovakia, including both premenopausal and postmenopausal subjects.

## 2. Subjects and Methods

This study is a part of cross-sectional survey conducted in Slovakia between 2009 and 2013 to analyze the effect of menopause on biomarkers of health in pre- and postmenopausal women. The entire study sample comprised 341 women ranging in age from 39 to 65 years (mean age = 49.11 ± 5.61). Of these, 259 participants provided all required data from the questionnaire and also anthropometrical, genetic, and biochemical data. The remainder (*n* = 82) failed to provide adequate information concerning at least one of these factors. Subjects were recruited from different localities in the western and middle parts of Slovakia via an invitation letter for the study circulated and distributed prior to data collection with the help of local medical doctors. Participants were then interviewed in a medical examination in the morning, and they were investigated with respect to their medical, anthropometrical, and life style aspects at local health centres. However, only selected variables were considered for the purpose of this paper. All participants gave written informed consent for participation in the study and they were always accompanied to their local health centre by trained anthropologists. 

Data concerning lifestyle habits including physical activity, smoking, and their health status and menstrual cycle characteristics were investigated by assisted questionnaire. 

Women were divided according to their menopausal status (MS) into pre- and postmenopausal groups, in accordance with the WHO definition [15].

All anthropometrical parameters were measured by professional anthropologists and the same instruments were used for all subjects. Anthropometric measurements were taken using standard anthropometric technique [16]. Blood pressure was measured in the morning, in the sitting position using a mercury sphygmomanometer, during a medical examination. 

Venous blood was collected after overnight fasting. The plasma was separated and biochemical analysis of gamma glutamyl transpeptidase (GMT), alanine aminotransferase (ALT), uric acid (UA), total cholesterol (TC), bilirubin (Bil), fasting blood glucose (Glc), total cholesterol (TC), TG, HDL-C, apolipoprotein A1 (apoA1), and apolipoprotein B (apoB) was carried out by routine laboratory methods in the Department of Clinical Laboratories of the Bratislava Alpha Medical. The LDL-C levels were calculated using the Friedewald formula [17]. The atherogenic indices were calculated as follows: apoB-to-apoA1 ratio, TC-to-HDL-C ratio, LDL-C-to-HDL-C ratio, log (TG-to-HDL-C ratio), and non-HDL-C as TC-HDL-C.

### 2.1. Genetic Analysis

Genomic DNA was extracted from peripheral blood samples using the SiMax Genomic DNA Extraction kit (Ecoli). PCR was used to detect the presence of the insertion (I) and deletion (D) alleles in intron 16 of the *ACE* gene, as previously described by Rigat et al. [18] and Danková et al. [19]. Genotyping of *CILP2* polymorphism (rs16996148 variant near CILP/PBX4 genes) was carried out by PCR-RFLP, as described in Rašlová et al. [5]. PCR product (135 bp) was cleaved by restriction enzyme Hin1II (Fermentas) and separated on 4% agarose gel (Super Fine Resolution (SFR), Amresco). The minor allele T is characterized by fragments of 82 and 53 bp, while an uncut fragment represents the major G allele.

### 2.2. Statistical Analysis

The Chi-square test was used to analyze whether the genotypic distribution and the alleles were in accordance with the Hardy-Weinberg equilibrium. An analysis of covariance was used to determine differences in variables over the menopausal status, *ACE* I/D genotypes, and *CILP2* genotypes, with age as covariate due to significant differences in age of women's groups. Analysis of covariance with age, body mass index (BMI), and waist-to-hip ratio (WHR) as covariates evaluated the relationship between the gene polymorphisms and biochemical parameters, and Bonferroni correction was used to test pairwise comparisons. Stepwise linear regression models were used for modelling the data. Only those variables that had values of *P* < 0.05 in the univariate analysis were included in the regression analysis as dependent variables. The values of age, BMI, WHR, TC-to-HDL-C, and LDL-C-to-HDL-C were not normally distributed and required logarithmic transformation. In addition, two-way analysis of variance was used to analyze a common effect of *CILP2* and *ACE* polymorphisms as risk factors on the values of LDL-C, and non-HDL-C. All statistical computations were performed with the SPSS 17.0 software program (SPSS Inc., Chicago, IL). A *P* value of less than 0.05 was considered statistically significant.

## 3. Results

The mean values of anthropometric, biochemical characteristics and blood pressure of women in the entire sample by menopausal status are shown in Table 1. As expected, postmenopausal women had lower mean values for bilirubin, HDL-C, and apoA1 and higher values for all other selected variables than the premenopausal ones. However, after adjustment for age these differences remained significant only for liver enzymes GMT and ALT.

The genotype distribution and allele frequencies of the *CILP2* gene polymorphism in the entire sample fell within the Hardy-Weinberg equilibrium (*χ* ^2^ = 0.07, df = 1, *P* > 0.05). The *CILP2* genotype and allele frequencies were as follows: GG = 90.6% (*n* = 299), GT = 9.1% (*n* = 30), TT = 0.3% (*n* = 1) frequency of the G allele = 95.2%, and T allele = 4.8%. The genotype distribution and allele frequencies of the *ACE* gene polymorphism in the entire sample did not fall within the Hardy-Weinberg equilibrium (*χ* ^2^ = 8.01, df = 1, *P* < 0.005). The *ACE* genotype and allele frequencies were as follows: DD = 35.4% (*n* = 111), ID = 41.4% (*n* = 130), II = 23.2% (*n* = 73) D allele = 56%, and I allele = 44%.

To address the association and impact of the *CILP2* polymorphism on CVD risk factors, we evaluated the mean values of anthropometrical and biochemical parameters on each genotype and tested the significance of differences between GG and GT/TT genotypes by ANCOVA. A statistically significant impact of particular genotypes on the investigated parameters (Table 2) was evident in the following variables: HDL-C (*P* = 0.007), LDL-C (*P* = 0.016), apoB (0.004), apoB-to-apoA1 ratio (*P* = 0.002), TC-to-HDL-C ratio (*P* = 0.005), non-HDL-C (*P* = 0.009), and LDL-C-to-HDL-C ratio (*P* = 0.006), even after adding the age, WHR, and BMI as confounding factors (Table 2). Here, higher mean levels of HDL-C and lower mean levels of the other investigated lipid parameters were registered in the GT/TT genotype carriers than in the GG carriers. Further, we tested the common effect of menopausal status and *CILP2* on lipid parameters. However, the two-way analysis of variance did not reveal a statistically significant interaction between these two risk factors and their common effect on lipids (*P* > 0.05). In addition, the Bonferroni pairwise comparisons shown in Table 3 confirmed the effect of *CILP2* genotype on the above mentioned parameters (*P* < 0.05). 

Table 4 shows differences in the mean values of particular variables between the II, ID, and DD genotypes of the *ACE* gene tested for significance by ANCOVA models. There was a statistically significant association between genotype and the following parameters: TC (*P* = 0.035), LDL-C (*P* = 0.027), and non-HDL-C (*P* = 0.023). The DD and ID carriers had significantly higher TC, LDL-C, and non-HDL-C levels than the II genotype carriers, even after adjustment for age, BMI, and WHR. In addition, we tested the common effect of menopause status and *ACE* on lipid parameters. However, the two-way analysis of variance did not reveal a statistically significant interaction between these two risk factors and their common effect on TC, LDL-C, and non-HDL-C (*P* > 0.05).  Table 5 shows the results of the Bonferroni pairwise comparisons. The difference in the TC, LDL-C, and non-HDL-C between the II and DD/ID genotype groups remained significant (*P* < 0.05), with lower estimated marginal mean values in II carriers than in the DD/ID groups. 

A stepwise regression analysis was used to test the independent impact of *CILP2* and *ACE* gene polymorphisms and other considered risk factors on the lipid and lipoprotein parameters (Table 6). The regression analysis confirmed the effect of *ACE* genotype on the TC, LDL-C, and non-HDL-C, as previously detected in ANCOVA models. Here, the II genotype and ID/DD genotype groups were compared and positive *B* coefficient was determined, indicating that the II genotype can lower serum levels of TC (*B* = 0.40), LDL-C (*B* = 0.37), and non-HDL-C (*B* = 0.41), respectively. In the same table, the stepwise regression analysis also confirmed the effect of *CILP2* genotypes (GT/TT versus GG) on LDL-C, non-HDL-C, HDL-C, apoB, and three atherogenic indices (apoB-to-apoA1, TC-to-HDL-C, and LDL-C-to-HDL-C). The positive values of estimated *B* coefficient indicated the lowering effect of the minor T allele on all lipid parameters, except for HDL-C. In this case, negative value of the *B* coefficient suggested an increasing effect of the T allele. Furthermore, a significant negative effect of current smoking on the above investigated lipid and lipoprotein parameters was observed (*P* < 0.05), except for the HDL-C levels. Menopausal status alone was not selected by the regression model as a risk factor for increased lipid levels.

Table 7 shows the common impact of *ACE* and *CILP2* genotype groups on mean values of the LDL-C and non-HDL-C cholesterol, respectively. The II and GT/TT carriers had the lowest mean values of both examined variables (LDL-C = 2.50 mmol/L; non-HDL-C = 3.01). On the other hand, the ID/DD and GG carriers had the highest mean values of both investigated parameters (LDL-C = 3.33 mmol/L; non-HDL-C = 3.97). 

**Figure 1:**
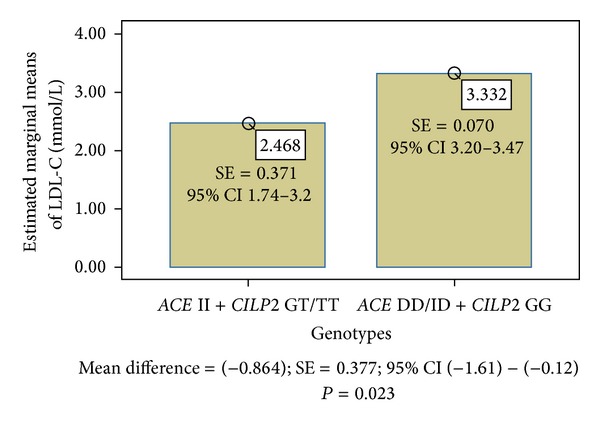
Association between *ACE*/*CILP2* genotypes and LDL cholesterol in Slovak women.

**Figure 2:**
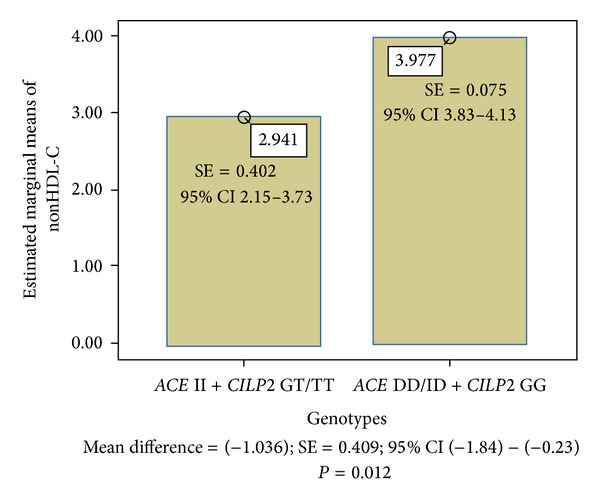
Association between *ACE*/*CILP2* genotypes and nonHDL cholesterol.

The association between the *ACE/CILP2* genotypes and level of LDL-C in Slovak midlife women is demonstrated in Figure 1. The *ACE/CILP2* protective variants (II + GT/TT) effect on LDL-C was significant in comparison with the *ACE* ID/DD carriers and homozygous carriers for *CILP2* G-allele (*P* = 0.023). A similar result was obtained for non-HDL-C in Figure 2. Here, the estimated marginal means of non-HDL-C were significantly different when the II + GT/TT carriers and DD/ID + GG carriers were compared (*P* = 0.012).

## 4. Discussion

In this study, we determined a profound impact of the *CILP2* gene on HDL-C, LDL-C, apoB, non-HDL-C levels, and three atherogenic indices in Slovak women. Only scanty and inconsistent information exists so far on associating this polymorphism with blood lipids. A relationship between *CILP2* gene polymorphism and TG and LDL-C concentrations was documented in European population, where the minor T allele was associated with lower concentrations of TG and LDL-C [1]. According to Járomi et al. [20], the relation of the *CILP2* gene to lipid metabolism is not yet discovered. The observations on the TG-lowering association were not replicated in Japanese population [21], in Hungarian population [20], or in the 40-years-old Slovak population [5]. Our study also failed to replicate the association between *CILP2* and TG concentrations. However, our results indicated that the minor T allele was associated with lower LDL-C, apoB, and atherogenic indices and higher HDL-C levels. In addition, Tai et al. [4] conducted a cross-sectional study which examined the relationship between *CILP2* gene polymorphism; blood lipid levels, and CVD prevalence in the Singaporean population ranging from 40 to 80 years of age. They found an association of the *CILP2* (T allele) with elevated HDL-C (*P* = 0.005) and lower LDL-C (*P* = 0.048) levels. Contrary to this finding, Zhuang et al. [22] did not observe a significant relationship between the *CILP2* gene and the serum lipid profile in the Japanese population. However, they investigated a lower frequency of T allele in patients with ischemic heart disease and 33% lower risk of the disease prevalence. Yan et al. [23] reported that the levels of TC, HDL-C, LDL-C, apoA1, and apoB in Han population (China) were associated with the *CILP2* genotypes in males but not in females. The inconsistent results in the above mentioned association studies could be caused by the different investigated populations and ethnic groups. Their exposure to different lifestyles and environments could modify the effect of these genetic variations on blood lipids. Different sample sizes could also play a role in the various findings.

When evaluating the impact of *ACE* I/D gene polymorphism on anthropometrical and biochemical parameters, we identified a statistically significant relationship with TC, LDL-C, and non-HDL-C in Slovak women. The DD/ID genotype carriers exhibited a worse lipid profile than the II carriers. Contrary to this finding, Cubrilo-Turek et al. [24] did not reveal statistically significant differences between the *ACE* DD/ID/II groups; the serum lipid, and apolipoprotein concentrations in Croatian menopausal women.

We have considered common influence of menopausal status and *ACE* I/D polymorphism on lipid parameters due to the fact that according to Proudler et al. [25] the serum *ACE* activity is modifiable, at least in part, by circulating levels of oestrogen and progestagen, which are levels that vary during menopausal transition. However, this effect was not confirmed in our study. 

In addition, the findings in this study showed a lack of association between the *ACE* genotype and blood pressure, and this is consistent with the previous studies in the Slovak population [19, 26].

In accordance with other studies [27–29], we found no evidence to suggest that the three *ACE* genotypes differ in BMI or WHR values. Moreover, Ryan et al. [30] suggest that total body fat mass, visceral and subcutaneous abdominal fat areas, plasma lipid levels, and systolic and diastolic blood pressures were not influenced by the *ACE* genotype in Caucasian and Afro-American women. Although, Bienertova-Vasku et al. [31] reported that the *ACE* I/D polymorphism did not express a prediction role on any of the investigated parameters of BMI, total body fat, total body water, waist circumference, hip circumference, WHR, and total body fat in Czech population, Das et al. [32] found that combined APOE∗4/4 and *ACE* DD genotypes had significant associations with elevated blood pressure, lipid abnormalities, and metabolic syndrome in adult Asian Indians. 

## 5. Conclusion

The results of this study indicate that the minor T allele of the *CILP2* gene and the I allele of the *ACE* gene have a protective effect against elevated blood lipid and lipoprotein levels. 

## Figures and Tables

**Table 1:** Anthropometrical, biochemical variables and blood pressure in Slovak women by menopausal status.

Parameter	Total	Premenopause	Postmenopause	*P* ^a^
	*n* = 341	*n* = 194	*n* = 147	
Age (years)*	49.11 ± 5.61	45.78 ± 3.93	53.50 ± 4.33	**<0.001**
Weight (kg)*	73.30 ± 15.22	71.92 ± 14.92	75.12 ± 15.46	0.786
WC (cm)	85.79 ± 14.24	83.29 ± 14.63	89.08 ± 13.04	0.722
HC (cm)*	104.29 ± 10.76	102.85 ± 10.17	106.19 ± 11.26	0.804
BMI (kg/m^2^)*	27.42 ± 5.56	26.59 ± 5.42	28.50 ± 5.57	0.307
WHR	0.82 ± 0.08	0.81 ± 0.09	0.84 ± 0.07	0.602
GMT (*µ*kat/L)*	0.42 ± 0.43	0.34 ± 0.30	0.52 ± 0.54	**0.022**
ALT (*µ*kat/L)*	0.33 ± 0.20	0.29 ± 0.18	0.38 ± 0.22	**0.030**
UA (*µ*mol/L)	256.00 ± 65.88	243.88 ± 64.09	272.00 ± 64.98	0.075
TC (mmol/L)	5.43 ± 1.03	5.32 ± 0.92	5.58 ± 1.15	0.840
TG (mmol/L)*	1.41 ± 0.85	1.30 ± 0.71	1.55 ± 0.99	0.621

	*n* = 340	*n* = 193	*n* = 147	

Bilirubin (*µ*mol/L)*	8.88 ± 4.16	9.01 ± 4.30	8.72 ± 3.98	0.831
Glucose (mmol/L)*	5.01 ± 1.38	4.78 ± 0.69	5.33 ± 1.90	0.103

	*n* = 315	*n* = 180	*n* = 135	

HDL-C (mmol/L)	1.56 ± 0.42	1.57 ± 0.43	1.53 ± 0.41	0.141
LDL-C (mmol/L)	3.25 ± 0.95	3.17 ± 0.85	3.37 ± 1.06	0.734
apoA1 (g/L)*	1.71 ± 0.42	1.72 ± 0.51	1.69 ± 0.25	0.522
apoB (g/L)	0.94 ± 0.25	0.92 ± 0.24	0.97 ± 0.27	0.711
apoB-to-apoA1	0.57 ± 0.18	0.55 ± 0.17	0.59 ± 0.20	0.537
TC-to-HDL-C*	3.73 ± 1.14	3.61 ± 1.08	3.88 ± 1.21	0.176
non-HDL-C	3.89 ± 1.06	3.76 ± 0.94	4.07 ± 1.17	0.624
LDL-C-to-HDL-C*	2.26 ± 0.93	2.18 ± 0.88	2.37 ± 1.00	0.171
log⁡(TG-to-HDL-C)*⁡*	−0.09 ± 0.29	−0.13 ± 0.29	−0.05 ± 0.29	0.239

	*n* = 309	*n* = 186	*n* = 123	

sBP (mmHg)*	123 ± 16.94	120 ± 15.55	127 ± 18.22	0.581
dBP (mmHg)*	79 ± 11.32	78 ± 10.16	80 ± 12.72	0.508

WC: waist circumference; HC: hip circumference; BMI: body mass index; WHR: waist-to-hip ratio; GMT: gamma glutamyl transpeptidase, ALT: alanine aminotransferase; UA: uric acid; TC: total cholesterol; TG: triglycerides, HDL-C: high density lipoprotein cholesterol; LDL-C: low density lipoprotein; apoA1: apolipoprotein A1; apoB: apolipoprotein B; sBP: systolic blood pressure; dBP: diastolic blood pressure. Values represent mean ± SD. *Not normally distributed parameters; ^a^adjusted for age.

**Table 2:** Anthropometrical, biochemical variables and blood pressure according to *CILP2* genotypes in Slovak women.

Parameter	*CILP2* genotype					Menopause status ∗ *CILP2 *
	GG	GT/TT	*F*	*P* ^a^	*P* ^b^	*P*
	*n* = 299	*n* = 31				
Weight (kg)	73.35 ± 15.61	71.97 ± 12.05	0.303	0.583		
WC (cm)	85.66 ± 14.55	84.20 ± 11.42	0.479	0.489		
HC (cm)	104 ± 11.11	105 ± 8.66	0.012	0.911		
BMI (kg/m^2^)	27.47 ± 5.68	26.43 ± 4.56	1.203	0.274		
WHR	0.82 ± 0.08	0.80 ± 0.08	1.386	0.240		
GMT (*µ*kat/L)	0.40 ± 0.40	0.47 ± 0.39	0.910	0.341		
ALT (*µ*kat/L)	0.33 ± 0.20	0.37 ± 0.23	0.949	0.331		
UA (*µ*mol/L)	255 ± 65.55	256 ± 67.38	0.000	0.996		
TC (mmol/L)	5.45 ± 1.05	5.10 ± 0.83	3.629	0.058		
TG (mmol/L)	1.42 ± 0.89	1.17 ± 0.47	2.733	0.099		

	*n* = 298	*n* = 31				

Bilirubin (*µ*mol/L)	8.82 ± 4.13	9.58 ± 4.47	0.963	0.327		
Glucose (mmol/L)	5.02 ± 1.41	4.79 ± 0.82	1.032	0.310		

	*n* = 276	*n* = 28				*n* = 304

HDL-C (mmol/L)	1.54 ± 0.40	1.76 ± 0.53	7.810	**0.006**	**0.007**	0.882
LDL-C (mmol/L)	3.29 ± 0.96	2.84 ± 0.72	6.076	**0.014**	**0.016**	0.557
apoA1 (g/L)	1.70 ± 0.43	1.77 ± 0.27	0.689	0.407		
apoB (g/L)	0.95 ± 0.26	0.80 ± 0.18	9.289	**0.003**	**0.004**	0.821
apoB-to-apoA1	0.58 ± 0.18	0.47 ± 0.14	10.572	**0.001**	**0.002**	0.500
TC-to-HDL-C	3.77 ± 1.13	3.13 ± 0.93	8.601	**0.004**	**0.005**	0.304
non-HDL-C	3.92 ± 1.07	3.38 ± 0.81	7.497	**0.007**	**0.009**	0.485
LDL-C-to-HDL-C	2.29 ± 0.92	1.78 ± 0.77	8.454	**0.004**	**0.006**	0.352
log⁡(TG-to-HDL-C)	−0.09 ± 0.29	−0.18 ± 0.26	2.989	0.085		

	*n* = 269	*n* = 29				

sBP (mmHg)	122 ± 17.16	126 ± 16.07	0.956	0.329		
dBP (mmHg)	79 ± 11.54	79 ± 10.38	0.052	0.820		

WC: waist circumference; HC: hip circumference; BMI: body mass index; WHR: waist-to-hip ratio; GMT: gamma glutamyl transpeptidase, ALT: alanine aminotransferase; UA: uric acid; TC: total cholesterol; TG: triglycerides, HDL-C: high density lipoprotein cholesterol; LDL-C: low density lipoprotein; apoA1: apolipoprotein A1; apoB: apolipoprotein B; sBP: systolic blood pressure; dBP: diastolic blood pressure. Values represent mean ± SD. ^a^Adjusted for age; ^b^adjusted for age, BMI, and WHR.

**Table 3:** Bonferonni pairwise comparisons between *CILP2* genotypes and lipid levels.

Parameter	*CILP2* genotype	Estimated marginal mean	SE	95% CI			Mean difference	SE	*P*	95% CI for difference	
HDL-C	GT/TT	1.74	0.07	1.60	1.88	GT/TT versus GG	0.20	0.07	**0.007**	0.06	0.35
	GG	1.54	0.02	1.49	1.58						
LDL-C	GT/TT	2.84	0.18	2.49	3.19	GT/TT versus GG	−0.45	0.19	**0.016**	−0.81	−0.08
	GG	3.29	0.06	3.17	3.40						
apoB	GT/TT	0.81	0.05	0.72	0.90	GT/TT versus GG	−0.14	0.05	**0.004**	−0.24	−0.05
	GG	0.95	0.01	0.92	0.98						
apoB-to-apoA1	GT/TT	0.47	0.03	0.41	0.53	GT/TT versus GG	−0.11	0.03	**0.002**	−0.17	−0.04
	GG	0.58	0.01	0.56	0.60						
TC-to-HDL-C	GT/TT	3.18	0.20	2.79	3.56	GT/TT versus GG	−0.11	0.03	**0.002**	−0.17	−0.04
	GG	3.76	0.06	3.64	3.88						
non-HDL-C	GT/TT	3.39	0.19	3.01	3.77	GT/TT versus GG	−0.53	0.20	**0.009**	−0.93	−0.14
	GG	3.92	0.06	3.80	4.04						
LDL-C-to-HDL-C	GT/TT	1.81	0.17	1.48	2.14	GT/TT versus GG	−0.48	0.17	**0.006**	−0.82	−0.14
	GG	2.29	0.05	2.19	2.40						

Based on estimated marginal means.

^a^Adjustment for multiple comparisons: Bonferroni.

**Table 4:** Anthropometrical, biochemical variables and blood pressure according to *ACE* genotypes in Slovak women.

Parameter	*AC* *E* genotypes			DD versus ID versus II		II versus ID/DD			Menopausal status ∗ *ACE*
	DD	ID	II	*F*	*P* ^a^	*F*	*P* ^a^	*P* ^b^	*P*
	*n* = 111	*n* = 130	*n* = 73						
Weight (kg)	73.30 ± 15.91	72.86 ± 15.27	73.61 ± 15.10	0.062	0.940	0.024	0.877		
WC (cm)	84.36 ± 13.84	85.78 ± 14.95	87.38 ± 14.35	0.697	0.499	1.018	0.314		
HC (cm)	105 ± 11.54	104 ± 10.30	105 ± 11.20	0.549	0.578	0.347	0.556		
BMI (kg/m^2^)	27.24 ± 5.85	27.41 ± 5.56	27.56 ± 5.34	0.021	0.979	0.027	0.868		
WHR	0.80 ± 0.07	0.82 ± 0.09	0.83 ± 0.08	2.422	0.090	1.162	0.282		
GMT (*µ*kat/L)	0.41 ± 0.38	0.39 ± 0.35	0.42 ± 0.51	0.135	0.873	0.121	0.729		
ALT (*µ*kat/L)	0.34 ± 0.22	0.33 ± 0.20	0.30 ± 0.17	1.109	0.331	1.990	0.159		
UA (*µ*mol/L)	257 ± 69.37	254 ± 64.61	258 ± 66.42	0.096	0.908	0.031	0.860		
TC (mmol/L)	5.49 ± 1.03	5.50 ± 1.07	5.23 ± 0.97	2.189	0.114	4.393	**0.037**	**0.035**	0.377
TG (mmol/L)	1.49 ± 1.04	1.34 ± 0.68	1.43 ± 0.89	1.091	0.337	0.008	0.927		

	*n* = 110	*n* = 130	*n* = 73						

Bilirubin (*µ*mol/L)	8.62 ± 4.06	9.38 ± 4.80	8.53 ± 3.33	1.354	0.260	0.712	0.399		

	*n* = 111	*n* = 130	*n* = 72						

Glucose (mmol/L)	5.02 ± 1.38	4.98 ± 1.56	5.01 ± 1.00	0.070	0.933	0.008	0.928		

	*n* = 103	*n* = 118	*n* = 68						

HDL-C (mmol/L)	1.57 ± 0.41	1.60 ± 0.46	1.54 ± 0.38	0.393	0.676	0.480	0.489		
LDL-C (mmol/L)	3.27 ± 0.99	3.33 ± 0.95	3.03 ± 0.83	2.619	0.075	5.011	**0.026**	**0.027**	0.975
apoA1 (g/L)	1.73 ± 0.26	1.75 ± 0.59	1.62 ± 0.24	1.855	0.158	3.646	0.057		
apoB (g/L)	0.95 ± 0.25	0.95 ± 0.28	0.89 ± 0.20	1.603	0.203	3.172	0.076		
apoB-to-apoA1	0.57 ± 0.19	0.57 ± 0.18	0.56 ± 0.17	0.019	0.981	0.035	0.851		
TC-to-HDL-C	3.74 ± 1.14	3.69 ± 1.12	3.58 ± 1.10	0.548	0.579	0.981	0.323		
non-HDL-C	3.94 ± 1.09	3.93 ± 1.06	3.66 ± 0.96	2.268	0.105	4.549	**0.034**	**0.023**	0.951
LDL-C-to-HDL-C	2.26 ± 0.95	2.25 ± 0.88	2.12 ± 0.90	0.660	0.517	1.319	0.252		
log⁡(TG-to-HDL-C)	−0.08 ± 0.29	−0.12 ± 0.30	−0.10 ± 0.29	0.492	0.612	0.004	0.949		

	*n* = 98	*n* = 118	*n* = 70						

sBP (mmHg)	123 ± 17.77	123 ± 18.71	121.03 ± 13.65	0.781	0.459	1.509	0.220		
dBP (mmHg)	79 ± 9.41	79 ± 11.20	78.04 ± 14.56	0.260	0.771	0.451	0.503		

WC: waist circumference; HC: hip circumference; BMI: body mass index; WHR: waist-to-hip ratio; GMT: gamma glutamyl transpeptidase, ALT: alanine aminotransferase; UA: uric acid; TC: total cholesterol; TG: triglycerides, HDL-C: high density lipoprotein cholesterol; LDL-C: low density lipoprotein; apoA1: apolipoprotein A1; apoB: apolipoprotein B; sBP: systolic blood pressure; dBP: diastolic blood pressure. Values represent mean ± SD. ^a^Adjusted for age; ^b^adjusted for age, BMI, and WHR.

**Table 5:** Bonferonni pairwise comparisons between *ACE* genotypes and lipid levels.

Parameter	*AC* *E* genotype	Estimated marginal mean	SE	95% CI			Mean difference	SE	*P*	95% CI for difference	
LDL-C	II	3.02	0.11	2.79	3.24	II versus ID/DD	−0.29	0.13	**0.027**	−0.55	−0.03
	DD/ID	3.31	0.06	3.18	3.43						
TC	II	5.21	0.12	4.98	5.45	II versus ID/DD	−0.29	0.14	**0.035**	−0.56	−0.02
	DD/ID	5.50	0.07	5.37	5.63						
non-HDL-C	II	3.62	0.12	3.38	3.87	II versus ID/DD	−0.33	0.14	**0.023**	−0.60	−0.05
	DD/ID	3.95	0.07	3.81	4.08						

Based on estimated marginal means.

Adjustment for multiple comparisons: Bonferroni.

**Table 6:** Regression analysis of selected confounder effects on lipid, and lipoprotein levels, and atherogenic indices in Slovak women.

Dependent variable	Independent variable	*B*	SE	Beta	*P*	95.0% CI for *B*		Collinearity statistics tolerance
TC	ln age	1.72	0.52	0.19	0.001	0.70	2.73	0.99
*n* = 292	Current smoker	0.32	0.09	0.20	0.001	0.14	0.50	0.98
	*ACE*: II versus ID/DD	0.40	0.14	0.16	0.005	0.12	0.69	0.98
	*R* ^2^ = 0.085, adjusted *R* ^2^ = 0.075, SE = 1.008							
	Excluded variables: menopausal status, BMI, WHR, former sport activities, and recent sport activities, current smoker.							
LDL-C	ln age	1.25	0.51	0.15	0.016	0.24	2.26	0.99
*n* = 259	Current smoker	0.29	0.09	0.19	0.002	0.11	0.47	0.98
	*ACE:* II versus ID/DD	0.37	0.14	0.16	0.009	0.10	0.65	0.98
	*CILP2:* GT/TT versus GG	0.42	0.20	0.13	0.035	0.03	0.81	1.00
	*R* ^2^ = 0.086, adjusted *R* ^2^ = 0.071, SE = 0.924							
	Excluded variables: menopausal status, BMI, WHR, former sport activities, and recent sport activities.							
non-HDL-C	ln age	1.52	0.59	0.16	0.010	0.37	2.67	0.91
*n* = 259	WHR	2.39	0.84	0.17	0.005	0.73	4.05	0.91
	Current smoker	0.33	0.10	0.20	0.001	0.14	0.53	0.98
	*ACE:* II versus ID/DD	0.41	0.16	0.16	0.008	0.11	0.72	0.97
	*CILP2*: GT/TT versus GG	0.52	0.22	0.14	0.018	0.09	0.94	1.00
	*R* ^2^ = 0.136, adjusted *R* ^2^ = 0.119, SE = 1.009							
	Excluded variables: menopausal status, BMI, former sport activities, and recent sport activities.							
HDL-C	ln age	0.49	0.20	0.14	0.017	0.09	0.89	0.92
*n* = 282	ln BMI	−0.59	0.14	−0.28	<0.001	−0.86	−0.32	0.69
	WHR	−0.89	0.35	−0.17	0.010	−1.57	−0.21	0.67
	*CILP2*: GT/TT versus GG	−0.21	0.08	−0.14	0.009	−0.36	−0.05	0.99
	*R* ^2^ = 0.180, adjusted *R* ^2^ = 0.168, SE = 0.370							
	Excluded variables: menopausal status, former sport activities, recent sport activities, and current smoker.							
apoB	ln BMI	0.26	0.08	0.19	0.001	0.10	0.41	0.99
*n* = 282	Current smoker	0.05	0.02	0.12	0.038	0.00	0.09	1.00
	*CILP2*: GT/TT versus GG	0.14	0.05	0.15	0.011	0.03	0.24	1.00
	*R* ^2^ = 0.075, adjusted *R* ^2^ = 0.065, SE = 0.249							
	Excluded variables: age, menopausal status, WHR, former sport activities, and recent sport activities.							
apoB-to-apoA1	ln BMI	0.23	0.05	0.25	<0.001	0.13	0.34	0.99
*n* = 282	Current smoker	0.04	0.02	0.13	0.026	0.00	0.07	1.00
	*CILP2*: GT/TT versus GG	0.11	0.04	0.16	0.004	0.03	0.18	1.00
	*R* ^2^ = 0.106, adjusted *R* ^2^ = 0.096, SE = 0.172							
	Excluded variables: age, menopausal status, WHR, former sport activities, and recent sport activities.							
ln TC-to-HDL-C	ln BMI	0.37	0.10	0.24	<0.001	0.17	0.57	0.70
*n* = 282	WHR	0.71	0.25	0.18	0.005	0.21	1.20	0.70
	Current smoker	0.06	0.03	0.13	0.022	0.01	0.11	1.00
	*CILP2*: GT/TT versus GG	0.17	0.06	0.16	0.004	0.06	0.29	1.00
	*R* ^2^ = 0.182, adjusted *R* ^2^ = 0.170, SE = 0.273							
	Excluded variables: age, menopausal status, former sport activities, and recent sport activities.							
ln LDL-C-to-HDL-C	ln BMI	0.61	0.12	0.28	<0.001	0.37	0.85	0.99
*n* = 282	Current smoker	0.08	0.04	0.13	0.024	0.01	0.15	1.00
	*CILP2*: GT/TT versus GG	0.25	0.08	0.17	0.003	0.08	0.42	1.00
	*R* ^2^ = 0.124, adjusted *R* ^2^ = 0.115, SE = 0.395							
	Excluded variables: age, menopausal status, WHR, former sport activities, and recent sport activities.							

**Table 7:** The common impact of *ACE* and *CILP2* genotypes as risk factors on mean values of the LDL cholesterol and non-HDL cholesterol.

		Dependent variable				
Factor		LDL-C			non-HDL-C	
*AC* *E*	*CI* *LP*2	*n*	Mean	SD	Mean	SD
II	GT/TT	7	**2.50**	0.74	**3.01**	0.74
	GG	56	3.09	0.82	3.73	0.98
ID/DD	GT/TT	21	2.95	0.70	3.51	0.81
	GG	197	**3.33**	0.98	**3.97**	1.09
*ACE ∗ CILP*2			*P* = 0.553		*P* = 0.491	
